# Challenging beliefs: A review of the paradigm shift in the treatment of pectus excavatum from radical resection to minimally invasive bracing and non-surgical vacuum bell suction

**DOI:** 10.7196/AJTCCM.2020.v26i4.016

**Published:** 2020-12-01

**Authors:** I Schewitz, D Nuss

**Affiliations:** 1 Department of Cardiothoracic Surgery, Faculty of Health Sciences, University of Pretoria, Pretoria, South Africa; 2 Department of Surgery, Eastern Virginia Medical School, Norfolk, Virginia, USA

**Keywords:** Pectus excavatum, Minimally invasive repair, Nuss repair

## Abstract

In 1997, Nuss introduced a minimally invasive non-destructive procedure for pectus excavatum, which revolutionised the treatment of the condition. This review will give a brief history on the management of this condition, followed by a review of 1 034 cases that have been repaired from 2008 to 2018.

## Background

As endotracheal anaesthesia became more sophisticated in the first half of the 20th century, blood and fluid resuscitation became better understood, antibiotics were discovered and freely available, and surgical procedures got more advanced, radical and daring, culminating in heart transplantation in 1967. Thereafter, surgery suddenly did an about-turn and it became fashionable to make the smallest incisions possible and cause the least amount of collateral tissue damage using fibreoptic inspired laparoscopes and robots. The speed with which this advance in laser surgery occurred varied from specialty to specialty and procedure to procedure. With regard to thoracic malformations, especially pectus excavatum and carinatum, it took a long time for a paradigm to shift in the literature from advocating for open, wide and radical resection of all the offending tissue to minimally invasive procedures.^[1]^ In fact, Nuss *et al*.^[2]^ stated that it was not necessary to resect all the rib cartilages and isolate the sternum to correct the depression as the flexibility of the chest wall responded well to new bracing techniques, which required less time, resulted in minimal blood loss, and kept the rib cage intact with its normal flexibility and expansion during respiration.

## Historical progression

Chest wall deformities have been recorded by artists since ancient times in Egyptian tombs and by more recent artists such as Leonardo Da Vinci, who depicted a complex excavatum-carinatum deformity in 1510, more than 500 years ago. The first medical report was by Bauhinus and Schenk von Grafenberg in 1594, when they presented a case of a 7-year-old boy who was born with the sternum and ribs bent back towards the internal part of the chest and abdomen.^[3]^ The boy had difficulty breathing, appeared to be in danger of suffocating because of a viscid sputum and had a chronic irritating cough.^[3]^ Despite an excellent description of the deformity and associated symptoms by Bauhinus and Schenk von Grafenberg, nothing changed with regard to the treatment of these patients for the next 300 years as thoracic surgery remained 'off-limits' until endotracheal intubation became available in the 1920s. In 1931, Sauerbruch^[4]^ treated a young female patient with a severe pectus excavatum by performing bilateral partial rib cartilage resections and a sternal wedge osteotomy accompanied by external traction. The external traction was accomplished by passing a wire through the sternum which was brought out through the skin and attached to an orthopaedic traction system for 6 weeks to hold the sternum in place until the tissues were healed. In 1949, Ravitch^[5]^ modified Sauerbruch’s procedure by extending the resection of the cartilages and isolating the sternum. He stated that 'in order to do away with external traction, all the deformed cartilage needs to be removed and the intercostal structures transected so that the body of the sternum is free'.^[5]^ A very important point to note is that Ravitch did not only recommend wide and radical resection of the anterior chest wall structures, but he also recommended that the procedure be done in very young patients by stating that 'ample experience has demonstrated the ease and safety with which the operation can be performed, even in infants, and has convinced us that the younger the patient, the easier the operation for both the patient and surgeon and the more likely a restitution to a normal thoracic contour'.^[1]^ He recommended that in infants, operation is advised whenever the patient is seen.^[1]^ In 1961, Adkins and Blades^[6]^ recommended placing a bar behind the sternum after resecting all the cartilage and cutting the sternum free from all its attachments, and removing the bar after 6 weeks. The Ravitch procedure with or without the Adkins and Blades retrosternal bar became the standard procedure for pectus excavatum repair for more than 50 years.

Physicians began to question in the late 1990s whether wide resection in young children was the ideal way to treat this condition. Gellis^[7]^ recommended in his 'Paediatric Notes' that paediatricians should not refer patients for surgical correction. In fact, Martinez *et al*.^[8]^ had earlier raised alarms about the danger of asphyxiating chondrodystrophy in young children after the Ravitch repair and proved his hypothesis in an experimental study on baby rabbits. He stated that 'it appears necessary to develop alternative techniques that avoid the removal of costal cartilages and to re-evaluate the optimal age for repair of these malformations', but he did not make any suggestions as to how that should occur.^[8]^ In 1996, Haller *et al*.^[9]^ also drew attention to 'chest wall constriction after too extensive and too early operations for pectus excavatum'.^[9]^ As a result of these two publications, surgeons started to delay surgery until puberty.

**Fig. 1:**
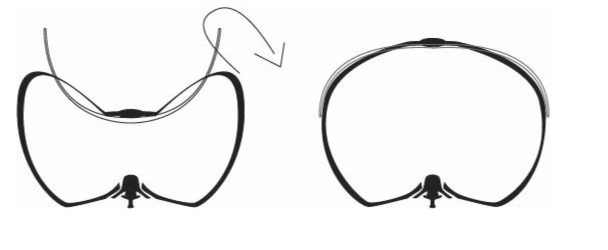
The correct shape of the Nuss bar is a semicircle which enters the pleural cavity just medial to the highest point

In 1987, Nuss performed a minimally invasive procedure for the repair of pectus excavatum that required no cartilage or sternal resection and used a sub-sternal bracing technique.^[2]^ He realised that the chests of young children are extremely flexible and noted the unsatisfactory outcomes of the wide resection technique. His procedure involved creation of a tunnel under the sternum, insertion of a convex titanium bar into the tunnel with the convexity facing posteriorly and then rotation of the bar by 180° thereby 'popping' the chest wall out of its depressed position (Fig. 1). The bar was removed 3 years later during an outpatient procedure. The procedure yielded excellent cosmetic results combined with normal chest expansion on inspiration.^[2]^

**Fig. 2:**
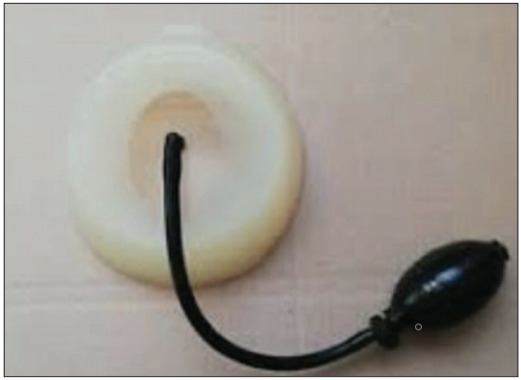
The suction device for pectus excavatum.

In 2003, Bahr presented a preliminary study on the treatment of pectus excavatum using a suction cup developed by Klobe, at the 1st World Congress of Paediatric Surgery, and the suction cup was later renamed the vacuum bell.^[10]^ Since then, there have been numerous studies showing that the vacuum bell is successful at correcting the defect in young patients with malleable chests, who are motivated to use it every day for 2 hours for at least 1 year.^[10]^ A single centre study by Obermeyer *et al*.^[11]^ reported good results on the use of the vacuum bell therapy in patients who are less than 11 years of age, have a symmetric deformity that is mild to moderate in severity and have a defect that is less than 1.5 cm deep (Fig. 2).

The indications to repair the defect are clear but the open repair has major complications and the cosmetic results are often inadequate. The Nuss procedure was an attempt to develop a reversible procedure that was less invasive, reduced complications and yielded better cosmetic outcomes

## Pathophysiology and symptoms of pectus excavatum

Pectus excavatum is the most common congenital abnormality of the chest wall in most parts of the world. Pectus carinatum is more common in Argentina than excavatum, emphasising the congenital element. The defect is thought to be caused by the anterior cartilage of the ribs pushing the sternum out (carinatum) or in (excavatum). The majority of cases are idiopathic. There is an increased incidence of pectus excavatum in people with Marfan’s syndrome.

The symptoms of pectus excavatum include exercise intolerance, especially for endurance sports, chronic tiredness, chest pains during exercise or rest, cardiac arrythmias, frequent chest infections and coughing. This condition is also associated with asthma.^[2]^ The psychological effects of pectus excavatum are under-appreciated and severely affect the quality of life of affected patients. These patients are mainly males who are typically introverted and stooped. They usually avoid sports or activities that will make the chest defect visible.

## Discussion

The principles of the minimally invasive procedure are deceptively simple, but it can have disastrous outcomes when poorly performed.^[12]^ Therefore, it is essential that surgeons are properly trained and mentored before attempting the procedure as the bar may injure the heart or lungs if improperly placed and not adequately stabilised

Since the publication of the minimally invasive procedure developed by Nuss in 1998, there has been a significant rise in the number of young patients who have been referred to surgeons by paediatricians or who have self-referred if their primary care physicians refused to do so. The number of patients who were treated in the Chest Wall Clinic at the Children’s Hospital of the Kings Daughters in Norfolk, Virginia, dramatically increased from 42 patients in 1987 - 1996 to 2 378 patients who presented for an evaluation and 1 173 patients who underwent surgery in 1997 - 2008.^[13]^ This phenomenon of a sudden influx of pectus excavatum patients also occurred in chest clinics around the world, as a total of 11 000 minimally invasive pectus excavatum repairs were performed in seven centres as reported at the Chest Wall International Group (CWIG) congress in Hong Kong in 2015 (personal communication by Nuss in July 2015). The influx of patients enabled physicians to develop management algorithms, protocols to do cardiac and pulmonary function studies and to review results in a reasonable timeframe, which in turn resulted in multiple modifications of the technique and instruments used for the repair.

### Modifications of the minimally invasive technique

The first modification was redesigning the bar from a short rectangular titanium bar to a steel bar that was much stronger and had curved ends.^[15,16]^ Other modifications included moving the incision from the anterior chest to the lateral chest wall, using thoracoscopy to facilitate tunnelling and bar placement, developing instruments specifically designed for the procedure, preventing bar displacement by adding a stabiliser to the bar on the left side, placing pericostal sutures around the bar and underlying ribs on the right side, elevating the sternum before tunnelling using a variety of techniques which included first tunnelling one or two interspaces superior to the deepest point and leaving the introducer in place to keep the sternum elevated while tunnelling under the deepest point, using the vacuum bell to elevate the sternum while tunnelling,^[16]^ and using Park’s Crane technique^[17–19]^ or by lifting the sternum with a retractor.^[20–22]^

Postoperative pain management has gone through several phases, the first phase being the use of standard narcotic medications used for pain control in most postoperative patients, starting in the recovery room after the patient was awake. However, this was found to be inadequate and thoracic epidurals were introduced and inserted by the anaesthetist in the operating room before the procedure was started. This proved to be very successful in many centres but the risk of spinal cord injury and the difficulty of epidural catheter insertion, prolonged operating room time, occasional misplacement of the epidural catheter and complex postoperative management caused several centres to switch to continuous, intravenous, and patient-controlled analgesia pumps initiated in the operating room before the patient was awake.^[23]^ Recently, cryo-analgesia has been introduced with excellent results and has allowed patients to be discharged on the 2nd or 3rd day after surgery with minimal postoperative pain and no long-term sequelae.^[24]^ Intercostal nerve blockers (bupivacaine) combined with scheduled oral narcotics and non-steroidal anti-inflammatory drugs administered immediately after the procedure have also allowed the patients to be discharged a day after surgery.^[25]^

### Cardiac and pulmonary function studies

Recent technical improvements and an increase in the number of patients seeking assistance in medical centres have permitted cardiac and pulmonary function studies to be performed to shed light on the pathophysiology of this condition. These studies have helped to explain why these patients almost universally state that they have more energy, are able to exercise more vigorously and for a longer duration of time after the Nuss procedure.^[14]^ Patients with severe pectus excavatum who are candidates for repair undergo cardiac analysis that includes computed tomography scans, magnetic resonance imaging, trans-oesophageal echocardiography and cardiac output studies. These studies have shown that there is considerable right heart compression with decreased filling and decreased stroke volume that is immediately corrected when the sternum is elevated and the pectus excavatum is corrected.^[27–29]^

A pulmonary function study (spirometry, plethysmography, oculoelectronic plethysmography, imaging studies and exercise testing) conducted on a large cohort of patients showed that the forced vital capacity, forced expiration volume in one second and forced expiratory flow are shifted to the left and after correction, the results are shifted to the right by one standard deviation.^[30]^ Moreover, oculo-electronic plethysmography revealed that the affected area of the chest does not move with respiration but after the Nuss repair, the chest movement is similar to normal controls.^[30]^

### Complications

In a review of 1 215 patients who were treated at the Norfolk Chest Wall Centre, it was reported that in the very early days of the Nuss procedure, the bar was not strong enough and that the bar was removed too soon, resulting in recurrence in 1.4% of the patients (n=11/1 215) that were treated from 1987 to 2008.^[13]^ In the recent review of 1 034 patients that were treated in the same centre from 2008 to 2018, there were no reports of recurrences after the introduction of the stabiliser and pericostal sutures (personal communication received from Kelly, 31 July 2019). Infection occurred in 1.4% (n=17/1 034) of the patients and the majority of these patients responded to antibiotics while 24% (n=4/17) of these patients required drainage and none required bar removal. Allergy to nickel was identified in 10.2% (n=105/1034) of the patients and all but 6 patients were identified preoperatively and received a titanium bar. One-third of the patients (n=2/6) who were unidentified to have a nickel allergy were successfully treated with prednisone and 66.7% (n=4/6) required bar removal, 16.7% (n=1/6) was switched to a titanium bar, and 50% (n=3/6) required no further treatment. A haemothorax occurred in 0.097% (n= 1/1 034) of the patients from a bleeding intercostal vein during the first 24 hours, which ceased spontaneously on chest tube drainage. Finally, 0.19% (n=2/1 034) of patients had a haemothorax secondary to trauma at a later date.

## Results

In a review of cases done at the Children’s Hospital of the Kings Daughters in Norfolk from 1987 to 2013, a total of 3 836 patients were evaluated for a chest wall deformity and 50% (n=1 921/3 836) were judged to be severe enough to warrant surgical repair. More than one-third of the patients (n=1 346/3 836) had already had their bars removed and 88.9% (n=1 197/1 346) of these patients were judged to have excellent results, 9.6% (n=129/1 346) had good results and 1.5% (n=20/1 346) had poor or failed results.^[22]^ More than a quarter of the patients (n=364/1 346) were adults aged 18 - 34 years and 272 of these patients already had their bars removed. Adult patients had better results than the younger patients, as they were operated on at a later date after the early learning period was over. Of the 272 adult patients that had their bar removed, 89.7% (n=244/272) had excellent results, 9.5% (n=26/272) had good results, 0.4% (n=1/272) had fair results and 0.4% (n=1/272) required a repeat of the procedure because of bar displacement.^[22]^

In a recent review from that same institution, of 1 034 patients that were repaired from 2008 to 2018, 48.6% (n=503/1 034) had bars and 92.6% (n=466/503) had excellent results, while 7.4% (n=37/503) had good results, demonstrating that the procedure is safe and reliable in experienced hands.^[30]^

## Conclusion

Innovators have constantly challenged the accepted beliefs of their day, created new paradigms and developed new procedures that have changed lives in positive ways.

The minimally invasive repair of pectus excavatum changed the paradigm from wide and radical resection of the anterior chest wall structures to a bracing technique that leaves the rib cage intact and requires no resection of any of the chest wall structures. Once it became clear that the chest wall is flexible and amenable to bracing techniques in young children, the age range was extended to include adults, with remarkably good results. In addition, young children with mild to moderate deformities can now be treated with non-surgical techniques such as the vacuum bell suction device. Pectus carinatum is being successfully treated with dynamic pressure braces and the small number of patients (10 - 20%) who fail bracing have the option of the minimally invasive Abramson procedure. These minimally invasive techniques and procedures have resulted in the return of normal cardiac and pulmonary function and excellent cosmetic results that are far superior to the mutilating resection procedures of the previous century. The minimally invasive procedures have been shown to be safe in centres that are experienced in various surgical techniques.
